# Neuropathophysiology, Genetic Profile, and Clinical Manifestation of Mucolipidosis IV—A Review and Case Series

**DOI:** 10.3390/ijms21124564

**Published:** 2020-06-26

**Authors:** Aleksandra Jezela-Stanek, Elżbieta Ciara, Karolina M. Stepien

**Affiliations:** 1 Department of Genetics and Clinical Immunology, National Institute of Tuberculosis and Lung Diseases, 01-138 Warsaw, Poland; jezela@gmail.com; 2 Department of Medical Genetics, The Children’s Memorial Heath Institute, 04-730 Warsaw, Poland; e.ciara@ipczd.pl; 3 Adult Inherited Metabolic Diseases, Salford Royal NHS Foundation Trust, Salford M6 8HD, UK

**Keywords:** mucolipidosis type IV, *MCOLN1*, corneal clouding, gastrin, myopathy, neurodegenerative

## Abstract

Mucolipidosis type IV (MLIV) is an ultra-rare lysosomal storage disorder caused by biallelic mutations in *MCOLN1* gene encoding the transient receptor potential channel mucolipin-1. So far, 35 pathogenic or likely pathogenic MLIV-related variants have been described. Clinical manifestations include severe intellectual disability, speech deficit, progressive visual impairment leading to blindness, and myopathy. The severity of the condition may vary, including less severe psychomotor delay and/or ocular findings. As no striking recognizable facial dysmorphism, skeletal anomalies, organomegaly, or lysosomal enzyme abnormalities in serum are common features of MLIV, the clinical diagnosis may be significantly improved because of characteristic ophthalmological anomalies. This review aims to outline the pathophysiology and genetic defects of this condition with a focus on the genotype–phenotype correlation amongst cases published in the literature. The authors will present their own clinical observations and long-term outcomes in adult MLIV cases.

## 1. Introduction

Mucolipidosis type IV (MLIV; #252650), is a rare autosomal recessive lysosomal storage disease (LSD) resulting from loss-of function mutations in the *MCOLN1* gene (*605248), which encodes for mucolipin-1 (ML1). This transient receptor, also known as potential channel protein mucolipin-1 (TRPML1), a vesicular Ca^2+^ release channel belonging to the transient receptor potential (TRP) superfamily [1,2,3], plays a role in endocytosis, and its dysfunction results in a build-up of lysosome substrates in the cytoplasm [4], aberrations in endosomal and autophagosomal trafficking, modifications to autophagy, abnormal regulation of lysosomal exocytosis, changes in the mammalian target of rapamycin complex 1 (mTORC1)/Transcription factor EB (TFEB) signaling axis, and dysregulation of heavy metal homeostasis [5].

Apart from TRPML1, the TRPML subfamily consists of two other members, TRPML2 and -3, which are encoded by *MCOLN2* and -3 genes, respectively. In humans, these proteins exhibit a common six-membrane-spanning topology. They interact in vesicular trafficking; their exact role in intracellular compartments is not completely understood yet. TRPML2 and TRPML3 have not been linked with any human disease [6].

Mucolipin-1 (ML-1) is a membrane protein consisting of six recognized transmembrane-spanning domains placed at late endosomes and lysosomes; a serine lipase domain and a nuclear localization signal [7,8]. Two proline containing sequences separate the first two transmembrane domains near the N-terminus, and a di-leucine motif (L-L-X-X) at the C-terminal tails regulate the protein trafficking to the late endosomal pathway [9,10,11]. Amino- and carboxyl-terminal tails have a cytosolic orientation, and the pore located between transmembrane segments 5 and 6, in the C-terminal half, contains Ser(557), the principal phosphorylation site, and Ser(559) [7,12,13].

The ML-1 channels may be involved in Ca^2+^ transport across membranes in lysosomes [14,15] in Ca^2+^-dependent fusion between the lysosomal and plasma membrane during the exocytotic stage of the membrane trafficking process, and in pathophysiological processes concerning lysosomal aggregation, proteolysis, and storage [15]. Lysosomal exocytosis has been shown to play a role in wound healing; membrane repair and cellular debris clearance [16]. Alterations in ML-1 and disturbance in these processes result in the accrual of heterogenous lipids and proteins in vacuoles in cytoplasm [13] and could be implicated in both corneal clouding [17] and achlorhydria [15,18], two hallmarks of MLIV.

### 1.1. The Mucolipin (TRPML) Subfamily

The mucolipins (TRPML) are a subfamily of channel proteins consisting of mucolipin 1, 2, and 3 (TRPML1, TRPML2, and TRPML3, respectively) [19,20].

The *MCOLN1* gene, located on chromosome 19p13.2–13.3, containing 14 exons and encoding a 580-amino acid potential channel proteinmucolipin-1 (TRPML1), was first described by Bargal et al. [1]. The protein is confined to the surfaces of late endosomes and lysosomes, in various tissues, with its highest expression documented in the brain, kidney, spleen, liver, and heart [21].

It has been shown that some members of the TPR channel superfamily are critical in sustaining satisfactory ion homeostasis, signaling, and membrane trafficking in endosomes by regulating the concentration of free calcium in cells and Ca^2+^ efflux from acidic milieu [22].

The regulation of autophagy has also been attributed to TRPML channels. It has been demonstrated in experimental studies that, when TRPML1 is absent in MLIV cells, the autophagy stimulation by TRPML3 is not regulated, resulting in the increased autophagosome formation, as previously shown in MLIV patient cells [7,23,24,25].

A recent study by Park et al. [26] has highlighted the major role of TRPML1 in guarding against involuntary, pathological fusion between the lysosomes and other intracellular organelles, including the secretory organelles [26]. Importantly, it may significantly impact future therapies of MLIV.

### 1.2. Ethnicity

Mucolipidosis type IV (MLIV) was first described in 1974 in an Ashkenazi Jewish (AJ) infant with congenital corneal clouding and systemic storage bodies [27]. Although 70%–80% of patients with MLIV are of Ashkenazi Jewish and Ashkenazi Polish origin [8], among whom the carrier frequency is reported to be 1 in 100 [8] and a predicted incidence is 1:42.000 [28], patients from all ethnicities have been reported to have similar symptoms and signs worldwide [12]. Identification of biallelic pathogenic variants in *MCOLN1* confirms the MLIV diagnosis.

To date, 35 different mutations in *MCOLN1* gene have been described in MLIV patients [29] (Supplementary Materials Table S1).

Most of these mutations lead to the creation of a nonfunctional protein or prevent any protein from being produced. Two mutations account for 95% of all MLIV cases in people with Ashkenazi Jewish ancestry [1,30]. The most common (founder) mutation (called major AJ variant), which accounts for 77%, is a splice site c.406-2A>G substitution and introduces a premature termination of the ML-1 translation. The other common mutation, which comprises 18%, is a large gene deletion c.-1015_789del called minor AJ variant. Approximately 60% of Ashkenazi Jewish patients with MLIV are homozygous for the major AJ pathogenic variant and >30% are compound heterozygotes for both (major AJ and minor AJ) pathogenic variants [31,32]. To date, only one homozygote for the minor AJ mutation has been identified [33]. The major AJ pathogenic variant occurs in only 6–10% of non-Ashkenazi Jewish individuals. It is uncertain how mutations in the *MCOLN1* gene result in such distinct (severe to mild) clinical manifestations in patients with MLIV.

## 2. Clinical Manifestations

Mucolipidosis type IV (MLIV) is a neurodevelopmental disorder, which in 95% of individuals manifests with typical findings, neurologic and ocular, with additional characteristic biochemical parameters. Pregnancy is usually uncomplicated, and infants with MLIV have generally normal development [34]. Children born at term have normal anthropometric parameters. Reduction of normal growth velocity has been previously documented [34]. Growth retardation has been evident in all patients, aged 20 months to 32 years, despite adequate food intake [35]. All these patients had feeding difficulties, failure to thrive, reduced facial movements, constant hypersalivation, and difficulties in swallowing, which according to Chitayat et al. [35], may have indicated cranial nerve involvement (Table 1).

In childhood and during the first two–three decades of life, the neurologic course of the disease is stable, akin to cerebral palsy; spastic diplegia or quadriplegia are frequent diagnoses [33,36].

Specific clinical and laboratory features are discussed in the article below.

### 2.1. Dysmorphic Features

Although rare, craniofacial dysmorphism and congenital anomalies have been previously described [12,35,36,37,38].

Riedel et al. (1985) observed mild facial dysplasia with full lips, tooth anomaly, and large, low-set ears, with prominent earlobes in a 17-year-old patient [37]. Another patient presented with “trigonocephaly with a narrow bitemporal diameter, a broad nasal bridge, bilateral epicanthic folds and esotropia, broad palatine ridges with a central ridge, prominent ears, bilateral clinodactyly of the fifth fingers, and partial cutaneous syndactyly of the 3rd and 4th toes” [35]. The patient reported by Chaer et al. (2018) was diagnosed with great vessel transposition, two palmar creases in the right hand, bilateral syndactyly of the second and third toes, facial dysmorphism (unspecified by the authors), hemangiomas on the neck and the calf, and a left occipital plagiocephaly (chromosomal microarray gave normal result) [38].

Interestingly, in 2020, Pode-Shakked et al. presented data on facial analysis to verify the hypothesis of recognizable facial features in MLIV [39]. Using the DeepGestalt algorithm [40], 50 two-dimensional facial images of 10 MLIV patients were analyzed and compared to age/sex-matched unaffected controls (Group A, *n* = 98) as well as to individuals affected with one of 100 dysmorphic syndromes, excluding ML-IV (Group B, *n* = 99). Assessments were computed and classification results described by a distribution curve, the receiver operating characteristic (ROC) curve and its respective area under the curve (AUC). The authors noted that the ML-IV cohort showed a mean AUC of 0.822 (*p*-value < 0.01) when compared to Group A and an AUC of 0.885 (*p*-value < 0.001) in comparison to Group B [40].

The facial phenotype of MLIV was nevertheless not specified by the authors. From our perspective and clinical experience with dysmorphic syndromes, the facial features typical for MLIV may consist of open-mouth appearance, a long face, which narrows with age, and puffy eyelids. Those, however, are not distinctive and overlap with characteristics of other genetic syndromes. Moreover, as clearly noted in Table 1, the features change with age, which makes a phenotypic diagnosis of MLIV very challenging. DeepGestalt technology seems thus to be beneficial for the elucidation and characterization of MLIV, as it has been already proved for other dysmorphic syndromes.

### 2.2. Neurological Findings

The clinical and neuroimaging findings suggest that the *MCOLN1* gene has a critical role in central nervous system development and in preserving neuronal integrity in the retina and cerebellum.

From a neurological perspective, significant psychomotor development delay (observed in early infancy), with hypotonia, tendon hyperreflexia, and lack of language development, are common features. The development usually never progresses beyond the age of 1–2 years [41]. Receptive language is, however, better than expressive. Affected children rarely achieve walking ability, and only some can sit unsupported [35]. In our clinical practice, all adult cases affected with MLIV are able to walk although myopathy has become more prominent with age (Table 1).

Epileptiform discharges consistent with cortical dysfunction have been described in 60% of individuals [35] but are rarely associated with convulsions [47]. It has been postulated that extensive white matter disease inhibits the dissemination of epileptiform discharges. The neurological course of the disease is not unique, and therefore it can be challenging to differentiate it from other neurodegenerative diseases. The clinical diagnosis of MLIV may be even more complicated in cases with mild neurological symptoms or additional, coexisting features such as deafness (see cases 1–3 in Table 1).

### 2.3. Myopathy

Progressive lower limb weakness and ataxia has been documented in some genotypes of MLIV [33]. Children described in this study were sitting or walking with support. Adult-onset myopathy has also been observed in our adult patients (see cases 1–3, Table 1).

Spasticity and hypotonia are common features in this group of patients. Tendon reflexes are symmetrical and brisk in them; with most having bilateral Babinski signs.

Hand usage was significantly impaired in 27 patients [33]. Four patients could finger-feed themselves with solid food, whereas pincer grasp was present in very few children. In general, repeated neurologic exam has shown a stable course of the disease in 70% of children. Those who presented with deteriorated motor function have been shown to develop cerebellar signs [33].

In addition, swallowing complaints reported by parents/caregivers ranged from mild to severe, with severity of complaints reported increasing for solid textures [35]. Coughing during and following meals was reported in 86% of patients. All subjects reported drooling of varying levels of severity.

The ptosis, myopathic face, gradual decline in facial movements, persistent hypersalivation, and difficulties in chewing are thought to be associated with cranial nerves palsy. Contrary to Chitayat et al. [35], Schell-Apacik et al. (2008) have reported that some MLIV patients (10%) may have hearing impairment [48].

### 2.4. Secondary Mitochondrial Dysfunction

Autophagic flux and autophagosome–lysosome fusion are defective in many LSDs, including MLIV [49,50]. Autophagic stress may cause accrual of damage organelles, including mitochondria, making the cells more predisposed to pro-apoptotic signals [51].

Mucolipidosis type IV (MLIV) fibroblasts have been shown to accumulate fragmented mitochondria and exhibit increased sensitivity to apoptosis induced by Ca^2+^-mobilizing agonists [47,52], and thus it has been postulated that modulation of autophagy might be an effective treatment mechanism for emerging therapies [5]. This approach has been recommended for the therapy of other neurodegenerative disorders [53,54]; autophagy induction with rapamycin has been shown to reduce neuronal death in animal studies on Huntington’s disease [55]. Strategies that enhance autophagy may be critical in the treatment development of LSD as well as utilizing therapies that maximize residual mitochondrial respiratory chain activities. However, since multiple cellular pathways are impaired in LSDs, therapeutic strategies to ameliorate oxidative stress, improve mitochondrial function, and enhance autophagy may prove beneficial in the treatment of these rare disorders.

Mucolipin-1 (ML-1) is part of an important feedback loop between mTOR and TFEB; both are connected by the mTORC1-mediated phosphorylation of ML-1 [56]. Phosphorylation of ML-1 [57] reduces its channel activity and leads to reduced activation of TFEB as a transcription factor [56].

ML-1 has been shown to play a role in iron homeostasis, and its deficiency therefore results in abnormal iron regulation observed in MLIV patients [18,58]. Specifically, TRPML1 is a non-selective cation channel and acts in Fe^2+^ trafficking in late endosomes and lysosomes. Disturbed iron homeostasis is likely to impair brain myelination and contribute to the hematological symptoms of MLIV patients [57]. Importantly, iron catalyzes the synthesis of reactive oxygen species (ROS), and it is suggested that this build-up of iron in ML-1-deficient cells is a possible explanation of neurodegeneration and mitochondrial degradation [59]. Furthermore, ML-1 activity regulating lysosomal zinc levels has been shown to cause zinc accumulation in lysosomes and in the brain tissue in ML-IV patients [60].

### 2.5. Brain Neuroimaging

Among 15 MLIV patients, the body of the corpus callosum was well structured, while the physiological increase of the genu or splenium was not present [61]. White matter abnormalities (the distribution of the hyperintensities included the periventricular, deep, and subcortical white matter in T2-weighted images, reduced signal intensity in the basal ganglia, and cerebellar atrophy, which is more prominent in older patients) have been described.

As a result, hypoplasia of the body of the corpus callosum with dysplasia/absence of the splenium and rostrum, and cerebellar atrophy have been described as pathognomonic neuroimaging characteristics of MLIV [44,56,57,58,59,60,61,62,63,64].

Increased T1-weighted signal and decreased T2-weighted sequences were consistent with ferritin deposition in the basal ganglia and thalami [33,61,65,66].

A localized cerebral neurodegenerative process in MLIV has also been observed in five MLIV patients by Schiffmann et al. [66]. During a 3-year follow-up, the neurological function remained unchanged with enhanced cortical and subcortical grey matter volumes and ventricular size, and decreased white matter and cerebellar volumes. White matter involvement was illustrated by both incomplete myelination and additional focal periventricular areas of demyelination. Diffusion-weighted imaging have shown abnormal parameters in all patients and in all brain regions examined; hence, Schiffmann et al. [66] suggested diffusion-weighted imaging as an adequate and sensitive biomarker for long-term monitoring of MLIV patients [66].

The mechanism of developmental brain abnormalities and insufficient myelination and additional focal periventricular areas of demyelination is not clear [46,63,67]. It has been shown, however, in the *Mcoln*1(−/−) knockout mouse model of MLIV that glial cell activation was enhanced in the brain, while myelination in cerebral and cerebellar white matter tracts was reduced [68]. Pathogenesis and animal studies have been extensively described elsewhere [12].

However, it has been postulated that the defective protein in MLIV is essential to cells at different developmental stages of the corpus callosum [61]. Cerebral and cerebellar atrophy suggestive of progression of the disease has been prominent in older patients. Frei et al. [61] have concluded that these changes may not be present at birth, but appear at approximately 4 years of age and becomes evident in the second or third decade of life [61].

MR spectroscopic imaging showed greater changes in neurons in the cerebellum and white matter compared to the basal ganglia and parietal cortex [69]. In addition, an autopsy case report has shown a reduction in numbers of neurons and astrocytosis were found in the thalamus, hippocampus (CA3), substantia nigra, basis pontis, inferior olivary nucleus, spinal cord ventral horn, and cerebellar Purkinje cell layer [42,67].

While only 15% of patients show a remarkable neurological symptom progression retinal dystrophy rapidly progresses [35], hypotonia, hyperreflexia, spasticity and dystonia can be apparent during the slow progression of this disorder [35]. Apart from the severe form of MLIV presenting in infancy, atypical forms with milder phenotypes with non-progressive neurologic deficits and minimal ophthalmic abnormalities and no significant neurological dysfunction, as well as additional features, have also been reported in MLIV [35,36,37,38,43,45,68,69] (Table 1). They often lead to late diagnosis [41,70].

### 2.6. Sleep Disturbance

Patients who were able to reach stage two sleep, had sleep spindles and vertex waves, which were not concurrent in time [71] and which implied a defect in myelination of the corpus callosum and thalamocortical projections in the white matter in the cortex [42,67,72].

### 2.7. Ocular Findings

As no striking recognizable facial dysmorphism, skeletal anomalies, organomegaly, or lysosomal enzyme abnormalities in serum are common features of MLIV, the clinical diagnosis may, however, be significantly improved because of characteristic ophthalmological anomalies.

Most 5-year-old, and older patients had a reduced visual ability [41], and the cause was multifactorial. Amir et al. (1987) analyzed ophthalmologic features among 20 MLIV individuals and concluded that visual deterioration is likely to be associated with retinal degeneration rather than with corneal clouding [34].

The corneal opacities resulting from the accumulation of phospholipids, mucopolysaccharides, and gangliosides in the epithelial cells [27] have been shown to be apparent between infancy and 5 years of age, and its presence at birth is rare [34]. As corneal clouding is the first noticeable sign, it is often an important diagnostic clue in MLIV [34,35]. While corneal clouding may be present at birth and progress over time, other clinical features may not be recognized for several years. Apart from corneal clouding, strabismus is most often the first sign [17] in 63% of children and became evident before 3 months of age in five children, 3 to 6 months in eight children, and 7 to 12 months in four children [17].

Affected individuals usually have corneal clouding or opacities of varying degrees but bilateral and symmetric gradual fibrous dysplasia and retinal degeneration with severe visual impairment have also been demonstrated by their early teens [37]. The remaining ophthalmic features include optic atrophy, cataract, ptosis, and ocular pain from corneal abrasion [17,34], esotropia, severe myopia, eye lid swelling, epiphora and ipsilateral facial flushing, conjunctival injection, photophobia, early cataract formation, and retinal vascular attenuation [34,35,37,73]. Abnormal eye movements, such as nystagmus or roving eye movements, have been documented in 23% of patients. whereas strabismus has been found in 54.5% of patients, with esotropia in seven, exotropia in four, and right gaze preference [17] in one patient. In single reports, MLIV manifested as isolated retinal dystrophy [74,75].

Chaer et al. (2018) demonstrated the first spectral domain optical coherence tomography (SD-OCT) imaging of abnormal corneal epithelium in a 2-year-old patient affected with MLIV [38]. The proband underwent ophthalmologic evaluation of possible eye movements because of prenatally diagnosed transposition of the great vessels and suspicion of vermis hypoplasia. It revealed pale optic nerves, arteriolar attenuation, and retinal pigmentary changes; hypoplastic optic nerves and a delayed P100 wave on visual evoked potentials (VEP) were noted. The corneal epithelium was hyperreflective, measured at 13.4 mm (normal, 3–4 mm), while the total central corneal thickness was 579 mm [38].

The electroretinogram (ERG) and visual-evoked potentials in the MLIV child have been shown to be normal [74]. In a different study, ERG gave evidence of the electronegative responses to a scotopic bright flash, which may suggest a disturbance in the inner segments of the photoreceptors, bipolar cells, or other middle retinal neurons [76].

### 2.8. Achlorydia

Achlorhydria and hypergastrinemia are hallmark findings of MLIV [12,18]. All MLIV patients have been documented to suffer from achlorhydria associated with secondary increases in blood gastrin at 1507 pg/mL (range 400 to 4100 pg/mL; normal 0 to 200 pg/mL) [33] (Table 1).

Achlorydia and frequent malabsorption of iron from food results in iron deficiency in MLIV patients [18,33,41,60,64,77]. Distended parietal cells containing large vacuoles with lamellar, concentric inclusions were identified on gastroscopy [17,18]. Elevated gastrin and cellular vacuolization have been confirmed in the *Mcoln1*−/− mice [13], which have been remarkably similar to those of MLIV patients [18].

The universal achlorhydria in patients with MLIV indicates that fully functional mucolipin is necessary for normal hydrochloric acid secretion [33]. Iron deficiency could be attributed to the alteration of Fe^3+^ absorption secondary to achlorhydria.

All MLIV patients had hypergastrinemia and showed chronic atrophic gastritis and enterochromaffin-like (ECL) cell hyperplasia on histologic evaluation of the stomach [77,78]. Atrophic changes, gastritis, and ECL cell hyperplasia have been observed even in the youngest patient. The severity of the inflammation in mucosae, chronic gastritis, and chronic atrophy identified on biopsies increased with age and were secondary to longstanding achlorhydria [78,79].

Frequent diarrhea has been documented in 38% and constipation in 5% of patients [34].

### 2.9. Skin

Definitive diagnosis of MLIV requires (unless by genetic testing) electron microscopic evaluation of cultured skin fibroblasts or a biopsy specimen obtained from the skin fibroblasts, corneal and conjunctival epithelial cells, conjunctival goblet cells, corneal keratocytes or in the cytoplasm of eccrine duct cells, and, to a lesser extent, of endothelial cells, skeletal muscle, and cerebral white matter [38,42,79,80]. Cultured skin fibroblasts from MLIV patients have been demonstrated to be autofluorescent [81].

Histopathologic findings consist of single-membrane limited cytoplasmic vacuoles containing both fibrillogranular material and membranous lamellae and concentric bodies forming concentric whorls [35,36,47,82,83]. Membrane-bound vacuoles have been shown to be present in endothelial cells, fibroblasts, and glandular epithelial cells, and membrane cytoplasmic bodies in glandular epithelial and in endothelial cells [84,85].

These intracellular amorphous materials represent moderate storage of mucopolysaccharides, phospholipids, and gangliosides [86], which explains heterogeneity in the material discovered within the lysosomes. Bach et al. (2005) have demonstrated that these macromolecules are transported into lysosomes at an increased rate, and not into the Golgi bodies to be recycled into new cell membrane [30]. Normal catabolism of these macromolecules is a possible explanation of the mild clinical features and organomegaly but the presence of slow neurodevelopmental deterioration [35].

In the cornea, the keratocytes epithelium is affected, whereas the endothelium, Bowman’s layer, and Descemet’s membrane have been shown to be spared [80].

### 2.10. Other Laboratory and Significant Clinical Findings

Altarescu et al. (2002) have observed that 46% of MLIV patients had iron deficiency, and 36% of them were anemic [33].

It has been shown that many adult patients (in their 20 s and 30 s) manifest renal disease or failure [5]. Thus, there is still a need to monitor and report MLIV individuals, especially older ones, to confirm our understanding of the life-long course of this disorder.

## 3. Discussion

The nonspecific or, rarely, isolated (i.e., the involvement of the epithelium seen on SD-OCT or hypergastrinemia) initial clinical and biochemical findings in MLIV and the lack of a non-invasive, specific diagnostic test (i.e., enzymology testing), most likely lead to misdiagnosis or delayed diagnosis of MLIV. In the recent past, the diagnosis was made by skin or conjunctival biopsy. Currently, access to genetic mutation analysis, including next-generation sequencing (NGS), allows the identification of mutations in the *MCOLN1* gene, confirming the clinical suspicion as well as genotypic correlation of the clinical features (Table 2).

Molecular analysis is the most effective diagnostic test, because it allows determination of the cause of the disease in 99% of affected individuals, regardless of age, disease severity, and phenotypic spectrum. Genetic testing for the two most common mutations is indicated, particularly in patients of Jewish ethnicity (allows for diagnosis in 95% of patients), but can commonly be negative in non-Jewish individuals. It is recommended that sequence analysis of *MCOLN1* is performed first, followed by gene-targeted deletion/duplication analysis in individuals with only one or no pathogenic variants. The NGS gene panel, which includes the *MCOLN1* gene, whole exome, or genome analysis, may also be considered, especially for patients with a high index of clinical suspicion of MLIV or an atypical clinical phenotype of this condition. The latter two methods allow for identification of both single nucleotide polymorphism (SNP) and copy number variants (indels, CNV), also in non-coding regions of the genes.

Regarding the genotype–phenotype correlation, it was proposed based on the location of the variants within the *MCOLN1* gene [41]. As reviewed in Supplementary Materials Table S1, variants in the loop between the first and second transmembrane domain resulted in a mild phenotype; severe presentation was, however, also observed (c.694A>C). In patients with variants in the third transmembrane domain, mild neurological manifestation with progressive retinal diseases was noted. The mildest MLIV phenotype results from variants in the fourth transmembrane domain, while mutations located between the fifth and sixth transmembrane domain were identified in patients with severe manifestation. Considering our adult patients (Table 1 and Supplementary Materials Table S1), they present severe phenotype of MLIV and, therefore, fall into the last category of variants. The causative variants (c.1256G>C) were localized in exon 11, TM4–TM5 loop, which were not previously described. Thus, we gave novel insight into the genotype–phenotype correlations in *MCOLN1*-related disease.

The sequence of symptom progression may vary in MLIV patients with developmental delay and deterioration in their vision in childhood, followed by deterioration in their speech and muscle function in early teens. Persistent iron deficiency anemia and constipation have been common complications in our adult patients. Sensorineural hearing loss, although not described before, was part of the clinical sequelae among all three adult patients.

## 4. Conclusions

The diagnosis of MLIV in children and adults proves to be challenging. The knowledge of classical and atypical presentations of MLIV helps correlate phenotype and genotype and assists in establishing the correct diagnosis. Currently, serum gastrin concentration and brain MRI play a vital role in the diagnostic pathway. A comprehensive molecular analysis of the *MCOLN1* gene is key to achieving the definitive diagnosis of MLIV and giving reliable genetic counselling to families at risk. Furthermore, advanced imaging methods, such as diffusion–tensor imaging, may play a role as “functional biomarkers” in the long-term follow-up of these patients. Currently, apart from supportive management, no therapy is available for this rare condition.

## Figures and Tables

**Table 1:** Reports of mild or atypical manifestation of MLIV.

Reference	Case and Age at Diagnosis	Gastrin Concentration	Skin/Conjunctival Biopsy Result	*MCOLN1* Genotype ^a^	Features
Riedel et al. 1985 [37]	23-year-old male and his brother	N/A	conjunctival biopsy: membrane-bound vacuoles filled with fibrillogranular material and concentric membranous lamellar bodies indicating both storage of mucopolysaccharides and complex lipids	N/A	no corneal opacities until teens age; facial dysmorphism (described in the text)
Amir et al. 1987 [34]	20 Ashkenazic Jewish patients (10F/10M) aged 2–17 years	N/A	typical inclusion organelles on electron microscopy of conjunctival biopsy and/or skin biopsy tissue. The diagnosis was confirmed by demonstrating abnormal gangliosides storage in cultured fibroblasts.	N/A	developmental delay, corneal clouding, language function reduced
Chitayat et al. 1991 [35]	5 patients aged 20 months to 32 years	N/A	skin and conjunctival biopsies	N/A	minor congenital anomalies and facial dysmorphism (1 case, described in the text); puffy eyelids (5 cases), ptosis; coarsening of the facial appearance; myopathic face, a decline in facial movement; delayed onset puberty and no associated growth spurt (1 case)
Casteels et al. 1992 [42]	16 years old girl	N/A	conjunctival biopsy	N/A	ophthalmological findings as the only features
Reis et al. 1993 [43]	16 years old girl	N/A	conjunctival biopsy	N/A	minor motor difficulties; mild psychological disturbances
Altarescu et al. 2002 [33]	28 patients (age range 2 to 25), age at diagnosis 1 to 14 years	1507 pg/mL (range 400 to 4100 pg/mL, normal 0 to 200 pg/mL)	skin or conjunctival biopsies and in cultured skin fibroblasts	c.[406-2A>G];[406-2A>G] p.?/p.? 9 pts	independent, ataxic, spasticity, reduced tone gait, modified pincer grasp (patient no 15); severe visual impairment as the only feature (patient no 13)
				c.[-1015_789del];[406-2A>G] p.0/p.? 8 pts	
				c.[-1015_789del];[-1015_789del] p.0/p.0	
				c.[304C>T];[1084G>T] p.(Arg102*)/p.(Asp362Tyr)	
				c.[406-2A>G];[1222_1224del] p.?/p.(Phe408del)	
				c.[1406A>G];[1406A>G] p.?/p.?	
				c.[-1015_789del];[473_474del] p.0/p.(Thr158Lysfs*25)	
				c.1406A>G];[514C>T] p.?/p.(Arg172*)	
				c.[1084G>T];[?] ^b^ p.(Asp362Tyr)/p.0	
				c.[1453_1463dup];[1453_1463dup] p.(Ser488Argfs*96)/p.(Ser488Argfs*96)	
				c.[317T>C];[1340T>C] p.(Leu106Pro)/p.(Leu447Pro)	
Bindu et al. 2008 [36]	4 cases; aged 14 (2), 16 and 18 years	N/A	electron microscopic studies of biopsy specimens from skin, conjunctiva, peripheral nerve, muscle and liver		no corneal abnormalities in all, optic atrophy in 1 patient; slowly progressive spastic paraparesis from the second decade of life (3 patients) and early childhood (1 patient); thin corpus callosum; intellectual disability (3 out of 4 patients); diagnosis of non-compressive myelopathy (1 case) (atypical presentation)
Tuysuz et al. 2009 [44]	Turkish patient	hypergastrinemia, iron deficiency anemia,	N/A	c.[1367C>T];[1367C>T] p.(Ser456Leu)/p.(Ser456Leu)	defects in the posterior limb of the internal capsule by MRI, cerebellar atrophy, micrognathia, and clinodactyly of the fifth fingers; spastic tetraplegia
Geer at al. 2010 [45]	4.5-year-old girl non-Jewish 11-year-old non-Jewish girl	710 pg/mL (0–99 pg/mL) 526 pg/mL (0–99 pg/mL)	skin biopsy: vacuoles containing granular material and lipids compatible with a lysosomal storage disease.	c.[236_237ins93];[694A>C] p.0?/p.(Thr232Pro)	developmental delay, hypotonia, language delays hypotonia, limb spasticity, severe hypoplasia of the corpus callosum
Mirabelli- Badenier et al. 2014 [46]	5-year old Italian male	191.26 pmol/L (11–54 pmol/L)		c.[395_397del;468_474dup]; [395_397del;468_474dup] p.?/p.? ^c^	postnatal growth deficiency, non-progressive psychomotor delay and spasticity at onset in infancy
Chaer et al. 2018 [38]	4-month-old French Canadian boy diagnosed at 2	726 ng/L (0–90 ng/L)	electron microscopy of skin biopsy: secondary lysosomes filled with concentric lamellar structures in the cytoplasm of eccrine duct cells and, to a lower extent, of endothelial cells	c.[694A>C];[785T>C] (p.Thr232Pro)/(p.Phe262Ser)	congenital anomalies and facial dysmorphism; hypoplastic optic nerves and a delayed P100 wave on visual evoked potentials.
Case 1	20 years Pakistani old female	157 pmol/L (<40 pmol/L)	N/A	c.[1256G>C];[1256G>C] p.(Arg419Pro)/p.(Arg419Pro)	GI: deranged liver function tests (ALT 123 U/L, ALP 1078 U/L), USS liver normal, fibroscan TE of 8 to 9 EkPa; gall bladder polyp; Eye: corneal clouding, cataracts, optic atrophy, retinal pigmentation; MRI head: subtle high signal in the periventricular deep white matter, the globus pallidus and thalamus signal slightly lower than in the rest of the basal ganglia on T2 sequences, suggestive of a relative increase in iron deposition; Hearing: sensorineural hearing loss, Other: severe learning disability, microcephaly, premature ovarian failure; recurrent periods of depression and anorexia
Case 2	23-year-old Pakistani female	179 pmol/L (<40 pmol/L)	N/A	c.[1256G>C];[1256G>C] p.(Arg419Pro)/p.(Arg419Pro)	GI: iron deficiency anemia at presentation; Developmental delay, severe learning disability; Eye: corneal clouding, optic nerve atrophy, retinitis pigmentosa, registered blind; MRI: small corpus callosum, but pituitary gland was unremarkable; Hearing: Sensorineural hearing loss Lost the ability to speak at the age of 8–10 year; CK 1400 U/L: congenital myopathy; premature ovarian insufficiency; MRI leg: osteofibrous dysplasia and adamantinoma
Case 3	27-year-old Pakistani male	198 pmol/L (<40 pmol/L)	N/A	c.[1256G>C];[1256G>C] p.(Arg419Pro)/p.(Arg419Pro)	GI: constipation, iron deficiency anemia; Eye: corneal clouding, retinitis pigmentosa, deterioration of his vision since the age of 5, nystagmus, exotropia, anterior and posterior subcapsular cataracts; Hearing: sensorineural hearing loss; Myopathy CK 3100 U/L; MRI: atrophic corpus callosum, microcephaly, deep white matter T2 signal abnormalities and hypointense thalami; Lost ability to communicate at the age of 15; Depression, gynecomastia

ALP—alkaline phosphatase; ALT—alanine transaminase; CK—creatine kinase; GI—gastrointestinal; MRI—magnetic resonance imaging; ^a^ The nomenclature of identified variants is described according to the Human Genome Variation Society guidelines (HGVS v 2.0, www.hgvs.org/mutnomen) and referral sequences to the cDNA.^b^ The mutation is heterozygous and appears in one parent only. However, the cDNA is homozygous to this mutation, indicating total absence of mRNA production from the other allele. ^c^ Double mutant allele—the allele resulted from 2 consecutive mutations *in cis* on the same allele: a microdeletion of 3 nucleotides (c.395_397delCTG) and a microduplication of 7 nucleotides (c.468_474dupTTGGACC) occurring in exon 3 and in exon 4, respectively.

**Table 2:** Diagnostic pathway of MLIV.

Pathway	Diagnostic Clues
primary clinical manifestations, sequence of symptoms	In the first year of life, bilateral corneal clouding and strabismus may be the first signs noted, prompting referral to the pediatric ophthalmologist and geneticist; By the age of 2 years, developmental delay and corneal clouding are evident (regression is not a characteristic); Neuroimaging, usually initiated by the psychomotor delay, may reveal the occurrence of cerebellar atrophy (progressive, evident in children older than 4 years), peculiar basal ganglia signal anomalies, marked corpus callosum hypoplasia, and white matter abnormalities played a crucial role in the differential diagnosis [46]; Feeding difficulties with growth failure are typical, described in patients from childhood to adulthood; of note: various degrees of developmental and motor delay are observed, but the neurologic status is stable; In early childhood, hypergastrinemia is noted; of note: gastrin level assessment may be of high diagnostic value, as a cost-effective evaluation of every child with global unexplained developmental delay (especially those who are hypotonic, non-ambulatory, and non-verbal [35]); Hypotonia, pyramidal tract signs, spastic quadriplegia, and severe dysarthria are among vital neurologic abnormalities; Dystrophic retinopathy, if it occurs, may be progressive; In approximately 50% of patients, iron deficiency occurs [17]; In adults, myopathy, spasticity, and impaired hand usage are observed; From the second/third decade, renal failure may be observed
diagnostic work-up	Suspicion based on clinical diagnosis (neurologic and/or ocular symptoms and brain neuroimaging) should be verified in skin or conjunctival biopsy to find characteristic lamellar and polymorphous cytoplasmic inclusions in the cells; Gastrin level assessment to prove hypergastrinemia (which is accompanied by constitutive achlorhydria); Genetic testing to identify pathogenic variants in *MCOLIN1*; Muscle biopsy (no value for MLIV recognition/confirmatory, only as differential diagnosis procedure)
molecular testing and family counselling	In Ashkenazi Jews two variants of *MCOLN1* (c.406-2A>G or c.-1015_789del) account for 95% cases; In total, 35 *MCOLN1* variants are known in MLIV patients; No genotype-correlation was documented; Intrafamilial clinical heterogenicity should be considered in genetic counselling; For at-risk families, with known genotypes, prenatal or preimplantation genetic testing (PGT) can be offered
follow-up	The clinical need for patients’ registry as crucial instruments to develop clinical research, to improve patient care and healthcare planning, to facilitate the planning of appropriate clinical trials, and to assess the feasibility of clinical trials
treatment	Currently, no cure or corrective management exist; therapy is symptomatic; of note: penetrating corneal graft surgery is ineffective; Modulation of autophagy (activation, with caloric restriction or treatment with rapamycin) might be an effective strategy for treatment [5,54]; Small molecule therapy using miglustat was shown to delay the cerebellar disease in the MLIV mouse model (but has not yet been tested in humans) [5]

## Supplementary Materials

The following are available online at https://www.mdpi.com/1422-0067/21/12/4564/s1, Table S1. Detailed data of MCOLN1 molecular variants used in Table 1.
