# Supplementary material for: Neuropathophysiology, Genetic Profile, and Clinical Manifestation of Mucolipidosis IV—A Review and Case Series
Source: Int J Mol Sci. 2020 Jun 26;21(12):4564. doi: 10.3390/ijms21124564 (PMC7348969; doi:10.3390/ijms21124564)
Supplement: Supplementary file 1 [file ijms-21-04564-s001.pdf]

Suppl Table 1. Detailed data of *MCOLN1* molecular variants used in Table 1.

cDNA and protein level	Gene and protein localization	Variant type and MAF [%][1]	Additional information	Pathogenicity prediction[2]	Database status[3]	Some phenotype correlation
c.-1015_789del<br>p.0	exon 1-7<br>null	gross deletion (minor AJ)<br>[no data]	Deletion of 6434 bps spans exon 1-6 and the first 12 bps of exon 7 and affecting the translation initiation codon (Met1). The consequence of this change is no protein product.	Functional analysis: Pathogenic (null mutation to yield no mRNA and no protein product).	HGMD accession: CG005059 (DM: Mucolipidosis IV)	Associated with a severe MLIV
c.236_237ins<br>p.0?	exon 2<br>null	inframe insertion<br>[no data]	The 93bp segment from mitochondrial NADH dehydrogenase 5 *(MTND5)* is inserted inframe prior to the last nucleotide of exon 2 of *MCOLN1*. This variation creates a frame shift starting at codon Gln79. The new reading frame ends in a STOP codon at position 8. Alteration abolish proper splicing of *MCOLN1*. The splice site at the end of the exon 2 is not used due to an inhibitory effect of the inserted segment, resulting in two aberrant splice products containing stop codons in the downstream intron and these products are eliminated via nonsense-mediated decay - NMD).	MutationTaster: disease causing<br>Functional analysis: Pathogenic (null mutation to yield no mRNA and no protein product).	HGMD accession: CN044483 (DM: Mucolipidosis IV)	Associated with moderate phenotype of MLIV
c.304C>T<br>p.(Arg102*)	exon 3<br>TM1-TM2 loop	Nonsense<br>[ALL:0.0024<br>AFR:0.0062<br>SAS:0.0033<br>NFE:0.0035]	The reading frame is interrupted by a premature STOP codon at position 102. The mRNA produced might be targeted for NMD.	SIFT: Deleterious<br>MutationTaster: disease causing<br>Predicted truncated protein or no product.	HGMD accession: CM003596 (DM: Mucolipidosis IV)<br>ClinVar: RCV000005443.5 (Pathogenic- Mucolipidosis type IV), RCV000498354.4 (Pathogenic - not provided).<br>dbSNP: rs121908373 (validated dbSNP entry - Clinical significance: CLIN_pathogenic).	Associated with a severe MLIV
c.317T>C<br>p.(Leu106Pro)	exon 3<br>TM1-TM2 loop	missense<br>[no data]	The substitution of the highly conserved amino acid. Moderate physicochemical difference between Leu and Pro. Variant decreases formation and extrusion of tubulovesicular structures when overexpressed; disrupts tetrameric assembly; abolishes lysosomal localization.	SIFT: Deleterious<br>MutationTaster: disease causing	HGMD accession: CM026007 (DM: Mucolipidosis IV)<br>ClinVar: RCV000194523.1 (Pathogenic - Mucolipidosis type IV)<br>Uniprot : this variant is reported as possibly pathogenic<br>dbSNP: rs797044825 (not validated dbSNP entry - Clinical significance: CLIN_pathogenic)	Associated with milder phenotype
c.395_397del<br>p.?	exon 3<br>TM1-TM2 loop	inframe deletion<br>[no data]	Microdeletion of 3 nucleotides CTG occurring in cis (on the same allele) with c.468_474dup	MutationTaster: disease causing	HGMD accession: CP149142 (DM: Mucolipidosis IV)	Associated with a severe MLIV

c.406-2A>G p.?	intron 3 null	splice site (major AJ) [ALL:0.019 AMR:0.0085 ASJ:0.41 NFE:0.0054 OTH:0.014]	Substitution affecting the splice site acceptor site in intron 3 leading to frame shift. The reading frame is interrupted by a premature STOP codon. The mRNA produced might be targeted for nonsense mediated decay (NMD).	MutationTaster: disease causing Predicted change at acceptor site 2 bps downstream: -100.0% (MaxEnt: -100.0%, NNSPLICE: -100.0%, SSF: -100.0%)	HGMD accession: CS002473 (DM: Mucolipidosis IV) ClinVar: RCV000825567.1 (Pathogenic- Mucolipidosis), RCV000058927.3 (Pathogenic - not provided), RCV000005438.7 (Pathogenic - Mucolipidosis type IV) dbSNP: rs104886461 (validated dbSNP entry - Clinical significance: CLIN_pathogenic)	Associated with a severe MLIV
c.468_474dup p.?	exon 4 null	frameshift duplication [no data]	Microduplication of 7 nucleotides TTGGACC occurring in cis (on the same allele) with c.395_397del. This variation probable creates a frame shift starting at codon Asn159. The new reading frame ends in a STOP codon at position 27.	MutationTaster: disease causing	HGMD accession: CP149142 (DM: Mucolipidosis IV)	Associated with a severe MLIV
c.473_474del p.(Thr158Lysfs*25)	exon 4 null	frameshift deletion [0.00]	Microdeletion of 2bps CC. This variation creates a frame shift starting at codon Thr158. The new reading frame ends in a STOP codon at position 25.	MutationTaster: disease causing	HGMD accession: CD003634 (DM: Mucolipidosis IV) ClinVar: RCV000192306.1 (Pathogenic - Mucolipidosis type IV) dbSNP: rs797044821 (not validated dbSNP entry - Clinical significance: CLIN_pathogenic)	No data
c.514C>T p.(Arg172*)	exon 4 null	Nonsense [0.00]	The reading frame is interrupted by a premature STOP codon at position 172. The mRNA produced might be targeted for nonsense mediated decay (NMD).	MutationTaster: disease causing	HGMD accession: CM003597 (DM: Mucolipidosis IV) ClinVar: RCV000195067.2 (Likely pathogenic - Mucolipidosis type IV) dbSNP: rs797044824 (not validated dbSNP entry - Clinical significance: CLIN_likely_pathogenic)	No data
c.694A>C p.(Thr232Pro)	exon 6 TM1-TM2 loop	missense [ALL:0.0016 NFE:0.0035]	The substitution of the highly conserved amino acid. Small physicochemical difference between Thr and Pro. Protein fails to localize to late endosomes; abolishes Fe(2+) permeability; disrupts tetrameric assembly; abolishes lysosomal localization.	SIFT: Damaging/ Tolerated MutationTaster (v2013): disease causing	HGMD accession: CM011400 (DM: Mucolipidosis IV) ClinVar: RCV000192300.2 (Likely pathogenic - Mucolipidosis type IV) Uniprot: this variant is reported as possibly pathogenic dbSNP: rs767122713 (Clinical significance: CLIN_pathogenic)	The typical, rather severe presentation MLIV

c.785T>C p.(Phe262Ser)	exon 7 TM1-TM2 loop	missense [no data]	The substitution of the highly conserved amino acid. Large physicochemical difference between Phe and Ser.	SIFT: Deleterious MutationTaster: disease causing	no data	Pathogenic variants found in the loop between the first and second transmembrane domain (87-298aa) possibly reduce the stability of mucolipin-1. Individuals with these pathogenic variants had a mild phenotype, an independent ataxic gait, and the ability to use their hands to feed themselves.
c.1084G>T p.(Asp362Tyr)	exon 9 TM3	missense [ALL:0.0008 NFE:0.0018]	The substitution of the highly conserved amino acid. Large physicochemical difference between Asp and Tyr. Variant affects channel activity; abolishes Fe($^{2+}$) permeability.	SIFT: Deleterious MutationTaster: disease causing	HGMD accession: CM003598 (DM: Mucolipidosis IV) ClinVar: RCV000727664.1 (Pathogenic - not provided), RCV000005442.7 (Likely pathogenic - Mucolipidosis type IV) Uniprot: this variant is reported as possibly pathogenic dbSNP: rs121908372 (validated dbSNP entry - Clinical significance: CLIN_likely_pathogenic)	This pathogenic variant was associated with mild psychomotor involvement; a slower progression of the retinal disease and a relatively mild neurologic phenotype, although membrane preparations containing mucolipin-1 with this pathogenic variant had no channel activity.
c.1222_1224del p.(Phe408del)	exon 10 TM4	inframe deletion [ALL:0.0004 NFE:0.0009]	Microdeletion of 3 bps TTC leads to the loss of residue Phe408, which is highly conserved amino acid in protein domain Polycystin cation channel, PKD1/PKD2. Variant does not affect channel activity; affects channel inhibition by low pH; still localizes to late endosomes.	MutationTaster: disease causing	HGMD accession: CD003635 (DM: Mucolipidosis IV) dbSNP: rs797044817 (not validated dbSNP entry - Clinical significance: CLIN_pathogenic)	Associated with the mildest MLIV phenotype
c.1256G>C p.(Arg419Pro)	exon 11 TM4-TM5 loop	missense [< 0.01]	The substitution of the highly conserved amino acid. Moderate physicochemical difference between Arg and Pro. This variant is in protein domain: Polycystin cation channel, PKD1/PKD2.	SIFT: Deleterious MutationTaster: disease causing	novel variant (this study)	No data
c.1340T>C p.(Leu447Pro)	exon 11 TM5	missense [no data]	The substitution of the highly conserved amino acid. Moderate physicochemical difference between Leu and Pro. This variant is in protein domain: Polycystin cation channel, PKD1/PKD2	SIFT: Deleterious MutationTaster: disease causing	HGMD accession: CM026008 (DM: Mucolipidosis IV) ClinVar: RCV000192526.1 (Pathogenic - Mucolipidosis type IV) Uniprot : this variant is reported as possibly pathogenic dbSNP: rs797044827 (not validated dbSNP entry - Clinical significance: CLIN_pathogenic)	Associated with a more severe MLIV

c.1367C>T p.(Ser456Leu)	exon 12 channel pore loop	missense [no data]	The substitution of the highly conserved amino acid. Large physicochemical difference between Ser and Leu. This variant is in protein domain: Polycystin cation channel, PKD1/PKD2	SIFT: Deleterious MutationTaster: disease causing Predicted change at acceptor site 8 bps upstream: -20.5% (MaxEnt: 0.0%, NNSPLICE: -41.0%, SSF: 0.0%)		Associated with a severe MLIV
c.1406A>G p.?	exon 12 channel pore loop, null	splice site [< 0.01]	The substitution of the highly conserved amino acid. This variant is in protein domain: Polycystin cation channel, PKD1/PKD2.	Substitution affecting the splice site acceptor site in intron 11 leading to frame shift. The reading frame is interrupted by a premature STOP codon. The mRNA produced might be targeted for NMD.	HGMD accession: CS003630 (DM: Mucolipidosis IV) ClinVar: RCV000192302.1 (Pathogenic - Mucolipidosis type IV). rs797044818 (validated dbSNP entry - Clinical significance: CLIN_pathogenic)	Atypical moderate form of mucolipidosis IV - affected individuals walk independently and have better communicative skills
c.1453_1463dup p.(Ser488Argfs*96)	exon 12 channel pore loop	frameshift duplication [< 0.01]	Microduplication of 11bps GGCCGCAGCAG. This variation creates a frame shift starting at codon Ser488. The new reading frame ends in a STOP codon at position 96.	MutationTaster: disease causing The mRNA produced might be targeted for NMD.	ClinVar: RCV000193040.2 (Pathogenic - Mucolipidosis type IV) dbSNP: rs1207178149 (validated dbSNP entry)	Associated with a severe MLIV

AJ - Ashkenazi Jewish; DM - disease mutation, MLIV - mucolipidosis IV, n/a - not applicable, NMD - nonsense mediated decay , TM - transmembrane domain.

[1]MAF - minor allele frequency in gnomAD public database (gnomad.broadinstitute.org) cluster ed with the major populations: [ALL: general population, AFR: African/African American, AMR - Admixed American, ASJ - Ashkenazi Jewish, OTH: other ancestry, NFE: Non-Finnish European, SAS: South Asian]

[2]Molecular variants were assessed by pathogenicity prediction tools: MutationTaster (www.mutationtaster.org) and SIFT (sift.bii.a-star.edu.sg) for missense changes localized in coding sequence and MaxEnt, NNSPLICE or SSF for nucleotide changes identified in splice-site, respectively.

[3]According database: *The Human Gene Mutation Database* (HGMD, www.hgmd.cf.ac.uk) , *ClinVar* aggregates information about genomic variation (ClinVar, www.ncbi.nlm.nih.gov), human single nucleotide variations (dbSNP, www.ncbi.nlm.nih.gov).
